# Poor social support as a risk factor for antenatal depressive symptoms among women attending public antennal clinics in Penang, Malaysia

**DOI:** 10.1186/s12978-017-0404-4

**Published:** 2017-11-02

**Authors:** Abdul Rashid, Rokiah Mohd

**Affiliations:** 1 Penang Medical College, Department of Public Health Medicine, Georgetown, Penang, Malaysia; 2 Penang State Health Department, Penang, Malaysia

**Keywords:** Antenatal, Depressive symptoms, Depression, Social support

## Abstract

**Background:**

Depression, a type of mental disorder which is portrayed by marked alterations in mood, is associated with distress and/or impaired functioning. Poor social support is an important risk factor for depression in pregnancy. An extensive literature search failed to show any published study conducted in Malaysia on antenatal depressive symptoms and the risk of poor social support on it. The aim of the study was to determine the risk of antenatal depressive symptoms due to poor social support.

**Methods:**

This cross sectional study was conducted among 3000 pregnant women attending antenatal clinics in Penang, Malaysia. Edinburgh Postnatal Depression Scale (EPDS) was used to screen for antenatal depressive symptoms and the Oslo-3 Social Support Scale (OSS-3) was used to measure social support. Odds ratio and adjusted odds ratio were used to quantify the risk of antenatal depressive symptoms due to poor social support.

**Results:**

The prevalence of depressive symptoms was 20%. Using OSS-3 scale to gauge social support, most of the participants had moderate support (61.3%) followed by poor support (22%) and strong support (16.7%). Social support was found to be significantly associated with depressive symptoms in this study (OR 2.2, aOR 2.1, AR 45%).

**Conclusions:**

Considering that an expecting mother’s psychological factors are important in the wellbeing of the mother and child, antenatal depression must be quickly identified. Screening pregnant women for social support can help identify women with higher risk of depression.

**Electronic supplementary material:**

The online version of this article (10.1186/s12978-017-0404-4) contains supplementary material, which is available to authorized users.

## Plain English summary

Pregnant women with poor social support are at higher risk for depression but despite the association with many health related events to both mother and child, antenatal depression is less studied compared to post-natal depression. An extensive literature search failed to show any published study conducted in Malaysia on antenatal depression and the risk of poor social support on it probably because like in other Asian cultures, social support is typically high in Malaysia. Pregnancy is generally considered an opportunity to expand the family lineage, so expecting mothers generally receive good physical, emotional and social support from family and friends.

Malaysia is undergoing extensive rural to urban migration with most young people moving to cities for better employment opportunities. As a result of this migration, there is a transformation from extended to nuclear family household system resulting in most expecting mothers to lose the traditional social support system and inadvertently losing the proven mechanism in easing, obtaining and maintaining psychological changes and preventing depressive symptoms during pregnancy.

This study was conducted among 3000 pregnant women who attended antenatal clinics in Penang, Malaysia to determine the risk of antenatal depressive symptoms due to poor social support. One in five participants had depressive symptoms and poor social support was significantly associated with depressive symptoms.

## Background

Depression, a type of mental disorder which is portrayed by marked alterations in mood, is associated with distress and/or impaired functioning [1]. Depression is projected to be the second leading cause of disability worldwide by 2020 and the fourth leading contributor to the global burden of disease [2]. Depression can range from mild, moderate to severe depression [3]. People with depression utilize more health care services, become a burden to caregivers, have decreased quality of life and are at risk for suicide [4].

Depression is more prevalent in women [5] and it has been reported that depression is the leading cause of disability adjusted life years for women globally [2]. Due to hormonal changes, women of child bearing age, particularly pregnant women, are at higher risk for depression [6–8]. Prevalence of antenatal depression has been reported as high as 20% [9, 10]. The prevalence rates reported for antenatal depression could be an underestimate considering that depressed women are less likely to participate in research due to fear, denial and stigma related to mental illness and probably because symptoms of depression could be mistaken for changes which normally occur during pregnancy, resulting in many women not seeking mental health services [11, 12]. Despite the high prevalence and high relapse rates reported for antenatal depression, less is known concerning factors affecting antenatal depression compared to post-natal depression [10, 13, 14].

Antenatal depression has been shown to have long lasting detrimental effect, not only on expecting mothers but also on their children and family [10, 15–17]. Although increased risks to adverse obstetrics outcome is controversial [18] some studies have shown an association of depression during pregnancy with poor attendance to antenatal clinics, intra uterine growth retardation, low birth weight, preterm delivery and failure to thrive in infants [9, 19–22] Women with antenatal depression are also at higher risk of preeclampsia and birth difficulties [9]. Depression during pregnancy has been linked to risk taking behaviours and unhealthy lifestyle habits including poor dietary habits leading to poor nutrition, smoking and illicit substance abuse [11, 23, 24]. Depression during pregnancy has been shown to be a predicator for postpartum depression [9, 13, 25] and can be a risk factor for depression during subsequent pregnancy’s [22, 26].

The risk factors for antenatal depression include genetic, environmental, psychological, biological and lack of social support [10, 27, 28]. Social support is important during the emotional and physical changes that occur in pregnancy [25, 29, 30] and it has been shown to be an important protective factor against depression. Lack of social support is an important risk factor for depression in pregnancy [13, 23]. Social support may be emotional or practical support which may be objective i.e. what is actually received or subjective i.e. what is perceived to have been received from partners, spouses, family members, friends, co-workers and neighbours etc. [31–33]. In this study social support is defined as the perceived support received from family members, husband/partner and friends. It is postulated that there is a correlation between perceived social support and monoamine activity in the brain [34–36].

In many Asian cultures including Malaysia, the perinatal period is valued because it is an opportunity to expand the family lineage and in general expecting mothers receive good physical, emotional and social support from family and friends [37]. However, Malaysia is undergoing extensive rural to urban migration with most young people moving to cities for better employment opportunities. As a result of this migration, there is a transformation from extended to nuclear family household system [38] resulting in most expecting mothers to lose the traditional social support system which usually comes from family members and inadvertently lose the proven mechanism in easing, obtaining and maintaining psychological changes during pregnancy and preventing depression in pregnant women [39].

Despite the association with many health related events to both the mother and child, antenatal depression is less studied compared to post-natal depression [12]. Early identification of antenatal depression provides the opportunity for the provision of best health care possible for both the mother and fetus in the primary health care setting [12, 14, 40]. Social support can be used to help identify women at higher risk of depression [30]. An extensive literature search failed to show any published study conducted in Malaysia on antenatal depressive symptoms and the risk of poor social support on it. Although research on antenatal depressive symptoms has been studied in other countries especially in the west, cultural differences may affect prevalence and associated risk factors [11, 22]. Complementing this literature with research from other contexts, such as Malaysia, is critical to ensuring the results are generalizable and reflect contextual differences. Although in general, social support is high in Malaysia especially during pregnancy but changing social structures in the country may cause a change in the social support level and may inadvertently affect depression in pregnancy. Because of these reasons the objective of this study was to determine the prevalence of antenatal depressive symptoms and the association of social support on antenatal depressive symptoms.

## Methods

### Study design

This cross sectional study was conducted among expecting mothers on follow up in the Ministry of Health’s antenatal clinics in Penang, Malaysia.

### Background place of study

The study was conducted in Penang, one of the 14 states in Malaysia. There are five districts in Penang with approximately 30 health clinics and 60 community clinics. The health clinics are staffed by Family Medicine Specialists or by Medical officers whereas the community clinics are staffed by the staff nurses or community nurses. Due to the limitation in terms of costs and time and considering that not all community clinics and health clinics provide all levels of antenatal services, only 20 health clinics in the state which had a Family Medicine specialist on staff were used for the study.

### Sampling

There were approximately 14,000 expecting mothers on antenatal follow-up in the state in 2015. All expecting mothers from the 20 selected health clinics irrespective of their gestational period and parity were included in the study. Stata was used to calculate the sample size. Using studies from Pakistan (25.0%) and Thailand (20.5%) [41, 42] as references, 2928 pregnant women would need to be enrolled in the study in order to determine 20% prevalence of antenatal depression with 90% power. Power of 90% was chosen to ensure higher probability of finding the estimated prevalence and because 90% power provides a sample size feasible within the study period. This study was conducted in the 20 health clinics that covered all five districts in the state. A random sampling method was used to select the participants. Every third expecting mother attending the antenatal clinics was invited to participate. Only women who could speak and understand Malay and English and provided informed consent were included in the study. Once the participant was interviewed, a colour-coded sticker was placed on the file to avoid duplication in the data collection process.

### Tool

The data for this study was acquired using a questionnaire which was tested for viability and feasibility prior to the start of the study by nurses working in a maternal and health clinic. The questionnaire was adapted to incorporate feedback from this initial phase. The participants were interviewed face to face in the antenatal clinics by 10 comprehensively trained nurses using a uniform protocol which was set up to minimize error and bias. The interviews were conducted by trained nurses in private in the nurse’s room to ensure privacy. The baseline demographic information collected included age, employment status and living arrangement. Race was categorized into three major races in Malaysia which included Malay, Chinese and Indian and all other races were categorized as others. The validated Malay and English versions of the Edinburgh Postnatal Depression Scale (EPDS) were used to screen for antenatal depressive symptoms. EPDS is an effective tool to screen for postnatal as well as perinatal depressive symptoms [43]. It consists of 10 items on how the participant felt during the past week. There are four response options, scored from 0 to 3 for each item. A score of ≥ 12 was used to define a case with depressive symptoms [44].

Social factors related to depressive symptoms assessed included participant’s perception of the support she received from husband/partner and family. The Oslo-3 Social Support Scale (OSS-3) was used to measure perceived social support. OSS-3 has never been used to study association with antenatal depression although it has been used for studies on the elderly in Malaysia [45, 46]. Similar Malay version of OSS-3 used in the elderly studies in Malaysia was used in this study. OSS-3 consists of three questions stated below with possible answers:

1. How many people are you so close to that you can count on them if you have a serious personal problem – none *(1)*, 1 to 2 *(2)*, 3 to 5 *(3)* and ≥ 5 *(4)*
2. How much interest and concern do people show in what you do?- a lot *(5)*, some *(4)*, uncertain *(3)*, little *(2)* and none *(1)*
3. How easy is it to get practical help from neighbours if you should need it?- very easy *(5)*, easy *(4),* possible *(3)*, difficult *(2)* and very difficult *(1)*

To reflect social support, the sum score which ranged from 3 to 14, was divided into three categories; 3 to 8 ‘poor support’, 9 to 11 ‘moderate support’ and 12 to 14 ‘strong support’ [47, 48]. The OSS-3 social support scale had a Cronbach’s alpha coefficient of 0.67.

### Analysis

Data was tabulated, cross tabulated and analysed using SPSS version 18 and Stata SE13. Prevalence of antenatal depressive symptoms is reported along with the odds of depressive symptoms between women who reported poor perceived social support and those who reported moderate or strong perceived social support. Attributable risk for the sample and population is also reported. Poor perceived social support as measured using OSS-3, family and husbands/partner support on the pregnancy were used in the logistics regression analysis to account for confounders and results were reported as adjusted odds ratio. The data used for analysis is available as Additional file 1. 

### Ethics

The research received the ethical approval form the Ministry of Health ethics committee [NMRR-12-1337-14,430]. All respondents were provided with a patient information sheet which provided information concerning the study including the reason, benefits and the participant’s rights not to participate or to withdraw from the study at any time. Only after the participant had read the information sheet was she asked to give a written informed consent before starting the interview. The data is stored in the researcher’s office with access to the data only available to the principal investigator. A non-identifiable code was used to ensure the confidentiality of the respondents.

## Results

Out of the 3270 patients approached in the health clinics, 3000 agreed to participate and were screened using EPDS. A total of 600 (20%) pregnant women had depressive symptoms.

Table 1 shows the demographic profile of the participants. The age of the participants ranged from 16 to 50 years old with a mean age of 29. Most (85.7%) of the participants were in the less than 35 years old age group. Majority were Malays (76.5%) followed by Chinese (12.1%), Indians (9.7%) and of the other races (1.7%). Most were employed full time (62.9%) followed by those unemployed (35.5%) and living with partners (68.8%).

**Table 1:** Demographic profile and social support variables of the participants

Variables	Frequency	Percentages
Demographic variables		
Age		
< 35	2570	85.7
35–40	392	13.1
> 40	38	1.3
Race		
Malay	2294	76.5
Indian	292	9.7
Chinese	363	12.1
Others	51	1.7
Occupation		
Unemployed	1064	35.5
Full time	1888	62.9
Part time	48	1.6
Living arrangement		
Parents	462	15.4
In laws	447	14.9
Partner and/or children	2064	68.8
Institution	27	0.9
Social Support variables		
How supportive is husband/partner on pregnancy		
Not, less and fairly supportive	59	2.0
Supportive	944	31.5
Very supportive	1997	66.6
Is the family supportive on the pregnancy		
No	78	2.6
Yes	2922	97.4
OSLO		
Poor support	661	22.0
Moderate support	1831	61.3
Strong support	500	16.7

Concerning the social support variables, most of the husbands/partners in this study were very supportive (66.6%) or supportive (31.5%) of the pregnancy and only 2% of them were not, less or fairly supportive. Overwhelmingly the families of the participants were supportive of the pregnancy (97.4%). Using the OSS-3 scale to gauge social support most of the participants had moderate support (61.3%) followed by poor support (22%) and strong support (16.7%).

Table 2 shows the risk of depressive symptoms associated with the variables which were studied. Participants whose husbands/partners are ‘not, less and fairly supportive’ (OR 1.9 95% CI 1.1;3.5) and husbands/partners who are ‘supportive’ (OR 1.4 95% CI 1.2;1.7) are at higher odds of having depressive symptoms compared to participants whose husbands/partners are ‘very supportive’. Differences in age, race, religion, occupation and living arrangement were not statistically significant. Participants with poor social support, gauged using OSS-3, had about twofold higher odds of having depressive symptoms compared to participants with moderate and strong support (OR 2.1 95% CI 1.8;2.7).

**Table 2:** Risk of depressive symptoms due to the independent variables studied

Variables	Depression Symptoms n (%)	No Depressive Symptoms n (%)	OR (CI %)
Demographic variables			
Age			
< 35	523 (20.4)	2047 (79.6)	*Reference*
35–40	69 (17.6)	323 (82.4)	0.84 (0.63;1.10)
> 40	8 (21.3)	30 (78.9)	1.04 (0.48; 2.29)
Race			
Others	9 (17.6)	42 (82.4)	*Reference*
Malay	464 (20.2)	1830 (79.8)	1.18 (0.57; 2.45)
Indian	67 (22.9)	225 (77.1)	1.39 (0.64; 3.00)
Chinese	60 (16.5)	303 (83.5)	0.92 (0.43; 1.99)
Occupation			
Part time	9 (18.8)	39 (81.3)	*Reference*
Unemployed	218 (20.5)	846 (79.5)	0.96 (0.79; 1.15)
Full time	373 (19.8)	1515 (80.2)	0.89 (0.43; 1.88)
Living arrangement			
Parents	94 (20.3)	368 (79.7)	*Reference*
In laws	99 (22.1)	348 (77.9)	1.11 (0.81; 1.53)
Partner and/or children	399 (19.3)	1665 (80.7)	0.94 (0.73; 1.21)
Institution	8 (29.6)	19 (70.4)	1.65 (0.70; 3.89)
Social support variables			
How supportive is husband/partner on pregnancy			
Very supportive	361 (18.1)	1636 (81.9)	*Reference*
Supportive	221 (23.4)	723 (76.6)	1.39 (1.15;1.67)*
Not, less and fairly supportive	18 (30.5)	41 (69.5)	1.99 (1.13;3.50)*
Is the family supportive on the pregnancy			
Yes	581 (19.9)	2341 (80.1)	*Reference*
No	18 (24.4)	59 (75.6)	1.29 (0.77; 2.19)
OSLO			
Moderate and strong support	396 (16.9)	1943 (83.1)	Reference
Poor support	204 (30.9)	457 (69.1)	2.19 (1.79;2.67)

*statistically significant

As shown in Table 3, a binary logistic regression was conducted using social support variables, the model had an overall correct predicted percentage of 80.0% and Nagelkerke R square 0.031. There is a twofold higher odds of having depressive symptoms among those with poor social support..

**Table 3:** Social support factors as risks for Depressive symptoms

Variable	B	Wald	Sig	aOR	95% CI
OSLO					
Moderate and strong support (R)					
Poor support	0.77	57.758	< 0.001	2.16	1.77;2.64
Family supportive of pregnancy					
*Yes (R)*					
No	0.95	0.12	0.73	1.10	0.64;1.89
Husband/partner supportive of pregnancy					
Supportive and very supportive (R)	0.32	1.15	0.28	1.38	0.77;2.47
Not, less and fairly supportive					

## Discussion

### Prevalence

In the present study, 20% of the participants had depressive symptoms. In general antenatal depression has been reported to range from 10 to 20% in pregnant women [9, 10, 13, 26]. In one systematic review it was found that 18.4% of pregnant women were depressed during their pregnancy and 12.7% had an episode of major depression and 14.5% had a new episode of major or minor depression during pregnancy [49]. There is a wide range in the reported prevalence of depression in pregnancy, the reported prevalence varied depending on the country, study design and stage of pregnancy studied. In the United States of America (USA) the prevalence has been reported as low as 6.9% [26] and as high as 20% [50] and in São Paolo, Brazil the prevalence was reported as 19.6% [28]. In prospective studies, 33% of pregnant women had depressive symptoms in the USA [30] and 25% in pregnant women did in Canada [25]. The prevalence of depression has been reported higher among black (22%) and immigrant women (42%) compared to white women (7–8.6%) [51, 52]. Studies in Turkey found the prevalence of antenatal depression at about 27% [39, 53].In Asia the prevalence of antenatal depression has been reported higher among Pakistani women (48.4%) and aboriginal Pakistani women (31.2%) [52] compared to pregnant women in Hong Kong (6.4%) [37] and in Thailand (20.5%) [41].

The prevalence rates reported could be an underestimate because pregnant women may be less likely to participate in research and when they do they underreport their depressive symptoms [37] on purpose due to stigma, fear and denial [11, 12, 54] or simply because they may be unaware of the services available to them [55] or they may genuinely mistake symptoms of depression with normal pregnancy [12].

### Social support

In this study the risk of antenatal depressive symptoms was found to be significantly associated with poor social support. Studies have shown the importance of social support on maternal mental health [10, 26, 27, 56]. Pregnant women with poor social support are at higher risk for depression during current [13] and subsequent pregnancies [26]. Even perceived low social support can influence depressive symptoms during pregnancy [25]. Since depression during pregnancy may lead to postpartum depression [13], social support during perinatal period may have an influence on postpartum depression [22, 25, 57].

Similar to the results of the current study, a study among 896 pregnant women in Berlin found low social support as an important risk factor for depressive symptoms compared with women with medium and high social support [23]. Social support has been strongly associated with depressive symptoms in studies conducted in the USA [26, 30]. Black and Hispanic women in the USA with lower social support have higher rates of perinatal depressive symptoms compared to white women [29, 40, 51]. Similarly studies in Canada [58], Turkey [39, 53] and studies among Asians have found the association between poor social support and depression [7, 42].

Good social support protects against antenatal depression even after taking demographic variables into consideration [23, 25, 29, 59]. Psychosocial and emotional changes affect interpersonal relationships and play a role of stressors [28, 30] which contribute to depression. In a study to determine the temporal association between post-partum depression and intimate partner violence, found that social support was an independent protective factor for postpartum depression [60]. It has been reported that physical, verbal and sexual abuse and cultural restrictions including competing for affection while living in a joint family system may function as factors associated with depression during pregnancy in Asians [52, 61]. Interpersonal conflicts may result in poor emotional and instrumental support from spouses, family and friends resulting in the lack of social stability and social participation [11, 23, 30, 39] leading to depressive symptoms [23, 50, 56].

### Strengths and limitation

This is the first study of its kind in Malaysia which provides important and valuable information concerning the prevalence of antenatal depressive symptoms and the influence of social support on it. However the authors are cognizant of the limitations of this study, importantly the sampling method. Sampling all expecting mothers irrespective of their gestational period and parity failed to take into consideration the variation in the hormonal and social levels, and other changes which may impact on the depressive symptoms during the different stages of pregnancy. There are many other factors which are involved in antenatal depression, shown by the Nagelkerke R square value in the regression analysis model, social support only explains a small fraction of the factors involved in the development of antenatal depressive symptoms. However understanding the role of social support is important in the Asian context which is taken for granted. The authors suggest a cohort study design involving pregnant women at different gestational ages followed through postnatally to determine the association of antenatal depressive symptoms with post-natal depression.

## Conclusions

Considering that an expecting mother’s psychological factors are important in the wellbeing of the mother and child [11, 15–17, 19–24, 27, 62], it is imperative that antenatal depressive symptoms are quickly identified. Poor social support can be a trigger to screen for depressive symptoms in pregnancy [12]. Screening pregnant women for social support can help identify women with higher risk of depressive symptoms [30, 63]. Because of the high cost associated with clinician interviews, some researchers have suggested using screening tools to identify depressive symptoms [13, 64]). All pregnant women attending the maternal and child health clinics should be screened for antenatal depressive symptoms by the nursing staff using the validated translated version of the EPDS. This screening can be followed with a more structured depression scale for women screened positive for the first screen [26].

## Additional file

Additional file 1: Data set for prevalance of poor social support as risk factor for antenatal depression. (SAV 28 kb)
